# Supplementary material for: Empirical methods for controlling false positives and estimating confidence in ChIP-Seq peaks
Source: BMC Bioinformatics. 2008 Dec 5;9:523. doi: 10.1186/1471-2105-9-523 (PMC2628906; doi:10.1186/1471-2105-9-523)
Supplement: Additional File 1 — A variety of html documents from the USeq web site detailing the available applications, their best usage, output file type descriptions, command line menus, etc. [file 1471-2105-9-523-S1.zip › USeqUserGuides/JavaDoc.zip!/JavaDoc/cgar/LabGroupUserType.html]

LabGroupUserType


|  |  |  |
| --- | --- | --- |
| **Overview** \| **Package** \| **Class** \| **Use** \| **Tree** \| **Deprecated** \| **Index** \| **Help** | |  |
| **PREV CLASS**   **NEXT CLASS** | **FRAMES**    **NO FRAMES**     **All Classes** |  |
| SUMMARY: NESTED \| FIELD \| CONSTR \| METHOD | DETAIL: FIELD \| CONSTR \| METHOD |  |


---


## cgar Class LabGroupUserType

```
java.lang.Object
  cgar.LabGroupUserType
```

---

``` public class LabGroupUserType extends java.lang.Object ```

For associating a lab group with a user type.

---

**Constructor Summary**

`LabGroupUserType(int labGroupId,
java.lang.String labGroupName,
int userTypeId)`


| **Method Summary** | |
| --- | --- |
| `int` | `getLabGroupId()` |
| `java.lang.String` | `getLabGroupName()` |
| `int` | `getUserTypeId()` |

**Methods inherited from class java.lang.Object**

`equals, getClass, hashCode, notify, notifyAll, toString, wait, wait, wait`

**Constructor Detail**

### LabGroupUserType

```
public LabGroupUserType(int labGroupId,
                        java.lang.String labGroupName,
                        int userTypeId)
```


**Method Detail**

### getLabGroupId

```
public int getLabGroupId()
```

---


### getLabGroupName

```
public java.lang.String getLabGroupName()
```

---


### getUserTypeId

```
public int getUserTypeId()
```


---


|  |  |  |
| --- | --- | --- |
| **Overview** \| **Package** \| **Class** \| **Use** \| **Tree** \| **Deprecated** \| **Index** \| **Help** | |  |
| **PREV CLASS**   **NEXT CLASS** | **FRAMES**    **NO FRAMES**     **All Classes** |  |
| SUMMARY: NESTED \| FIELD \| CONSTR \| METHOD | DETAIL: FIELD \| CONSTR \| METHOD |  |


---
